# Full-field laser-Doppler-vibrometry FRF dataset for a four-bolt aluminum plate under bolt-torque variation

**DOI:** 10.1016/j.dib.2026.113331

**Published:** 2026-10-12

**Authors:** Berkay Kullukcu, Dina Hannebauer

**Affiliations:** FG Machine Dynamics and Acoustics, TH Wildau, Hochschulring 1, Wildau, Brandenburg, 15745, Germany

**Keywords:** Bolted joint, Structural health monitoring, Vibro-acoustic measurements, Resonance tracking, Local frequency-response assurance criterion, Modal correlation

## Abstract

The dataset described in this article comprises full-field scanning laser Doppler vibrometry measurements from a four-bolt aluminum plate tested under controlled bolt-torque variation. Eighteen torque configurations span an all-tight reference, single-bolt intermediate (5 N m) and loose (0 N m) states, and multi-bolt loosening combinations. The repository contains 1838 pointwise Polytec PSV-500 H1 displacement-to-force FRF exports (919 amplitude and 919 phase files) over 1–51,200 Hz at 1 Hz spacing. Comparative analyses use 51 LDV scan points common to every torque state and both signal types. The package further provides scan-point and torque metadata, resonance-selection and tracking tables, global and local FRF-correlation products, recovered PSV acquisition metadata, acquisition-quality coherence/reference-force spectra, figures, and scripts for reproducing the processed layer. For this specimen, quantitative FRF/correlation analysis is recommended up to approximately 25 kHz; the higher-frequency portion is retained as raw data but is dominated by a comparatively low signal level.

Specifications Table

**Table:** 

Subject	Engineering & Materials science
Specific subject area	Structural dynamics, vibro-acoustic measurements, and laser-Doppler-vibrometry data for bolted-joint diagnostics.
Type of data	Pointwise FRF exports (TXT), acquisition-quality coherence and reference-force spectra (TXT), documentation and processed tables (CSV), images (PNG), scripts (PY), metadata (JSON/JSON-LD/CFF/YAML), text documentation (MD/TXT), packaged dataset (ZIP).
Data collection	A Polytec PSV-500 scanning laser Doppler vibrometer measured pointwise H1 displacement-to-force amplitude and phase while an automatic modal hammer fitted with a steel tip excited a fixed point near the lower-left plate edge, close to bolt 3. The 5 and 10 N m conditions were applied with a HAZET 5108B-3CT torque wrench (2.5–25 N m; manufacturer-specified accuracy ±3% of scale value); 0 N m denotes a fully loosened state. The plate rested on soft sponges to approximate free-free boundary conditions. One scan realization is archived for each of 18 nominal torque configurations. PSV 10.3.1.0 acquisition used FFT mode, three complex averages, 51,200 FFT lines, 128 kHz sampling, 1 s acquisition time, and 1 Hz resolution over 1–51.2 kHz. The vibrometer used a rectangular window and no acquisition-side filter; the force-reference channel used a force window (parameter 0.507) and no acquisition-side filter. Velocity response was integrated by PSV to displacement before H1 export.
Data source location	Institution: TH Wildau; City: Wildau; Region: Brandenburg; Country: Germany.
Data accessibility	Repository name: Zenodo Dataset name: Full-field laser-Doppler-vibrometry frequency-response-function data for a four-bolt aluminum plate under bolt-torque variation Data identification number: 10.5281/zenodo.22770267 Direct URL to data: https://zenodo.org/records/22770267
Related research article	B. Kullukcu, R. Pianowski, M.S. Özer, E. Altinsoy, D. Hannebauer, Screening bolt loosening in a four-bolt plate with global FRF correlation and local FRAC maps from full-field laser Doppler vibrometry, manuscript submitted for publication, 2026 [1]. The companion study analyzes only the all-tight reference and four single-bolt 0 N m cases; the additional 13 torque configurations are documented in this data article and repository.

## 1 Value of the Data

• The repository [2] provides 18 controlled bolt-torque configurations as raw pointwise H1 amplitude and phase FRFs, comprising 1838 TXT exports and a 51-point spatial subset common to all states at 1 Hz frequency resolution. The combination of broad torque-state coverage, spatially distributed measurements, and raw plus processed data layers provides a reusable benchmark for method development.
• The data can support FRF-based damage detection, resonance and modal-parameter identification workflows, MAC/FRAC and FRF-shape comparisons, bolt-condition classification, machine-learning method development, and vibration-based condition monitoring. They can also serve as experimental targets for independently developed numerical models, although no matching finite-element model is included in the repository.
• The four single-bolt 5 N m configurations provide an intermediate discrete torque condition between the corresponding 10 and 0 N m states. They support comparison among three nominal torque levels for each bolt, but they do not define a continuous torque-response or preload-response relationship.
• The related research article [1] uses only the all-tight reference and the four single-bolt 0 N m cases for resonance-group screening. This data article makes the remaining 13 configurations available for independent reuse: four single-bolt 5 N m cases, four one-tight/three-loose cases, four two-loose cases, and the all-loose case.
• Processed CSV tables, documented variables, checksums, scripts, software-environment files, and machine-readable metadata support direct reuse and independent reproduction of the post-processing. Recovered PSV acquisition settings and the quality-control coherence/reference-force spectra additionally allow users to assess excitation and response quality without inferring acquisition parameters from the H1 exports alone. The package can also be used in teaching and method development for experimental modal analysis, structural health monitoring, bolted-joint dynamics, and research-data management.

## 2 Background

The short communication [1] applies resonance-group screening to bolt-torque variation in a four-bolt plate and is supported by the data package [2]. The accompanying research article uses full-field laser Doppler vibrometry to compare an all-tight baseline against four single-bolt 0 N m cases using amplitude-only and phase-inclusive FRF correlation metrics together with local FRAC maps. The data article decouples the reusable measurements and data products from that focused interpretation and makes the additional 13 torque configurations available. The specimen is a generic laboratory demonstrator for plate/panel-type bolted connections relevant to rail-vehicle inspection and related mechanical assemblies; it is not intended as a geometrically scaled prototype of one specific aircraft, building, or machine joint. This simplified geometry is used to isolate preload-sensitive joint effects under controlled laboratory conditions.

Bolted joints are an established source of uncertainty in structural dynamics because preload controls contact stiffness, frictional dissipation, microslip, and local boundary conditions. Reviews and recent model-identification studies show that changes in bolt condition can shift resonance frequencies and reshape frequency-response functions, making well-documented benchmark datasets useful for testing damage indicators [[3], [4], [5], [6]]. Full-field laser Doppler vibrometry is relevant in this context because it captures the spatial redistribution of vibration over the measured surface rather than only at a few discrete sensor locations; continuous-scan and full-field LDV studies have demonstrated this advantage for modal identification and damage-sensitive response-shape analysis [[7], [8], [9]]. The processed tables in this dataset therefore connect the raw H1 amplitude and phase exports to established correlation-based tools such as MAC, FRAC, and FRF-shape damage localization [[10], [11], [12], [13]].

## 3 Data Description

The dataset consists of root documentation/metadata files and eight principal archive/data layers: 00_raw_exports, 01_documentation, 02_processed_tables, 03_figures, 04_scripts, 05_quality_control_coherence, 06_quality_control_force, and metadata. The original H1 raw-data layer contains 1838 tab-separated Polytec TXT exports: 919 amplitude files and 919 phase files covering 18 nominal torque states. Seventeen states contain 51 amplitude and 51 phase exports, whereas the all-loose state contains one additional exported scan point in each signal type. Comparative processing therefore uses the intersection of all point indices and retains the same 51 coordinates in every torque state and in both amplitude and phase data. The quality-control layer is split into two archives: 05_quality_control_coherence contains 919 pointwise coherence spectra, and 06_quality_control_force contains 919 pointwise Reference-1 force spectra covering the same 18-state inventory. Seventeen states contain 51 spectra of each quality-control signal, while the all-loose state contains 52; common-point quality comparisons use 18 × 51 = 918 point-state spectra. This harmonization avoids spatially mismatched comparisons and is documented by the scan-point metadata and processing scripts.

There are six graphs presented in the paper. Fig. 1 depicts the test setup. Fig. 2 represents the in-plane spatial mapping of the 51 common LDV points and the fixed impact location. Fig. 3, Fig. 4 present the median spatial-RMS spectra according to torque-state class for 1–12 kHz and 1–51.2 kHz, respectively; Fig. 4 additionally marks 25 kHz as the recommended upper limit for quantitative FRF/correlation analysis for this specimen. Fig. 5 describes the selected candidate peaks of the all-tight spatial-RMS spectrum. Fig. 6 provides example local FRF-correlation maps included in the processed-data folder. The raw-to-processed workflow is: (1) directly measured/exported H1 amplitude and phase TXT files; (2) point-index harmonization and, when needed, complex-FRF reconstruction after converting phase from degrees to radians; (3) derived spatial-RMS spectra, resonance candidates, retention decisions, tracking windows, tracked frequencies, global correlation metrics, discrete 10/5/0 N m torque-level tables, and family-wise summaries; and (4) figures and optional HDF5 output generated by the supplied scripts. The coherence spectra in 05_quality_control_coherence and Reference-1 force spectra in 06_quality_control_force form a supplementary acquisition-quality layer and are not inputs to the main H1 processing workflow. Thus, measured analysis quantities, acquisition-quality diagnostics, and derived quantities are distinguished explicitly.

**Fig. 1:**
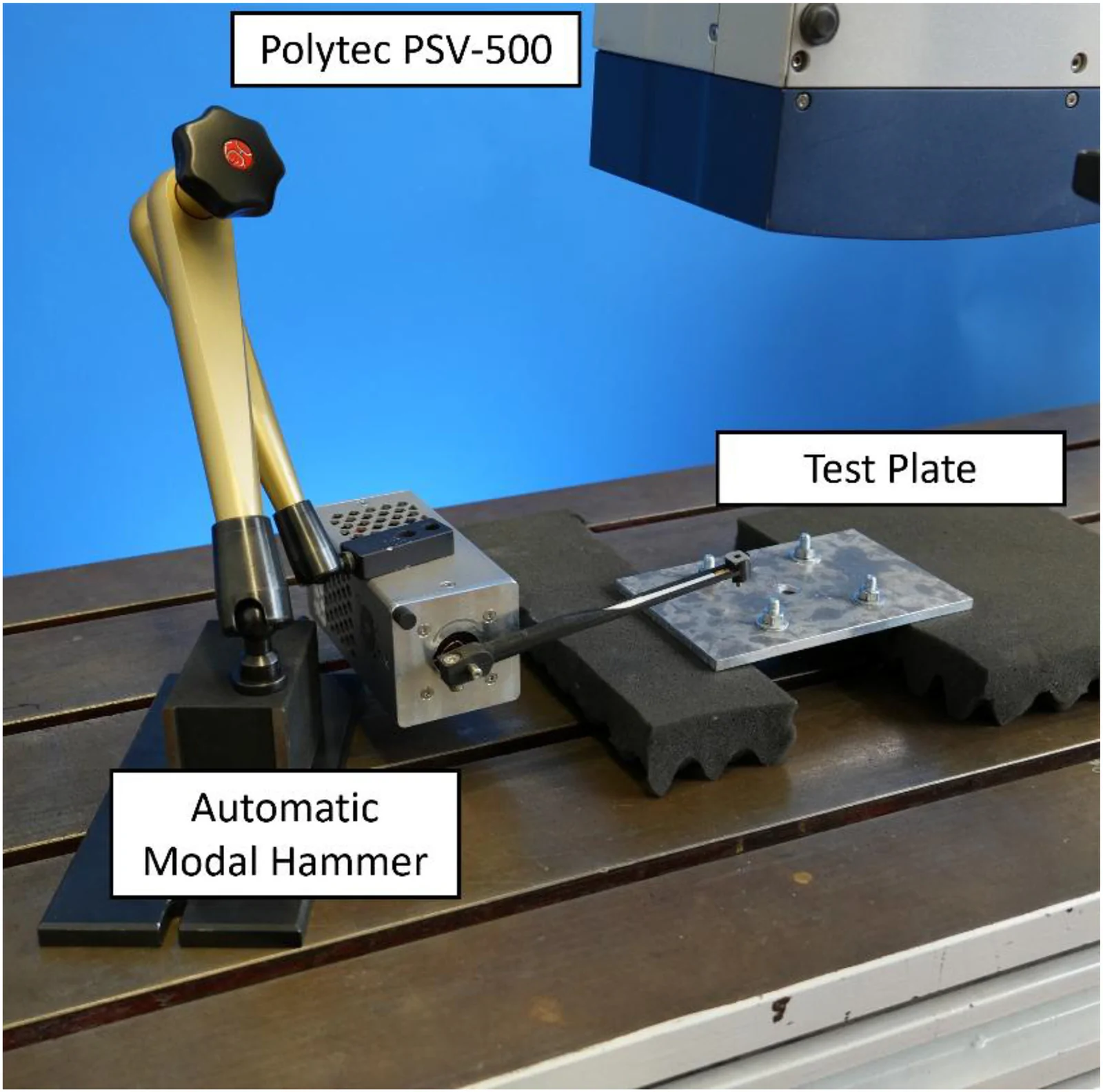
Experimental setup showing the Polytec PSV-500 scanning laser Doppler vibrometer, the four-bolt aluminum plate on soft supports, and the automatic modal hammer fitted with a steel tip used for repeated excitation.

**Fig. 2:**
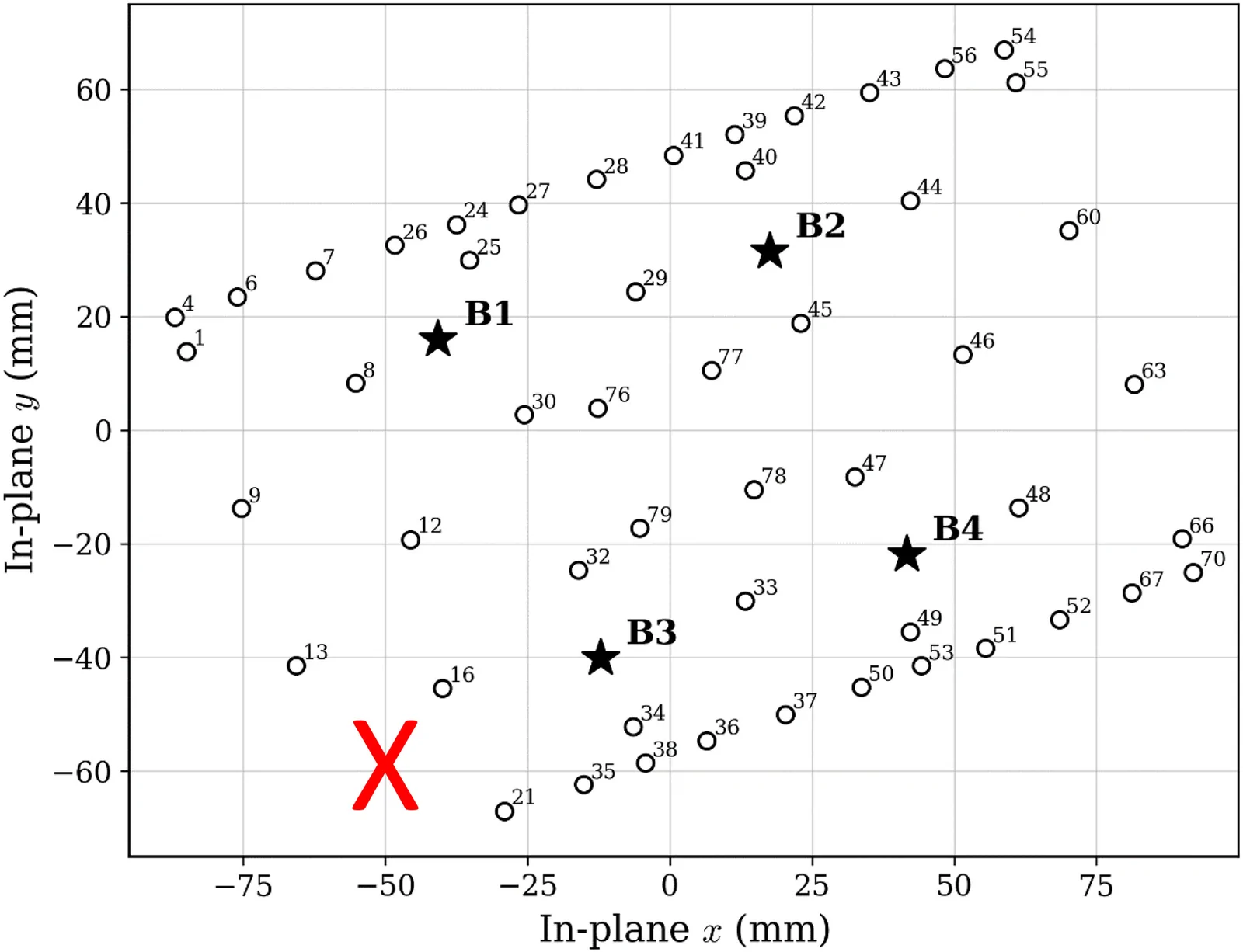
Spatial layout of the 51 LDV points (circles) common to all 18 torque states, projected onto the plate plane. Bolt centers B1–B4 are marked with stars. Numbers next to each point are the indices used in the raw export filenames. The red cross marks the impact point of the hammer.

**Fig. 3:**
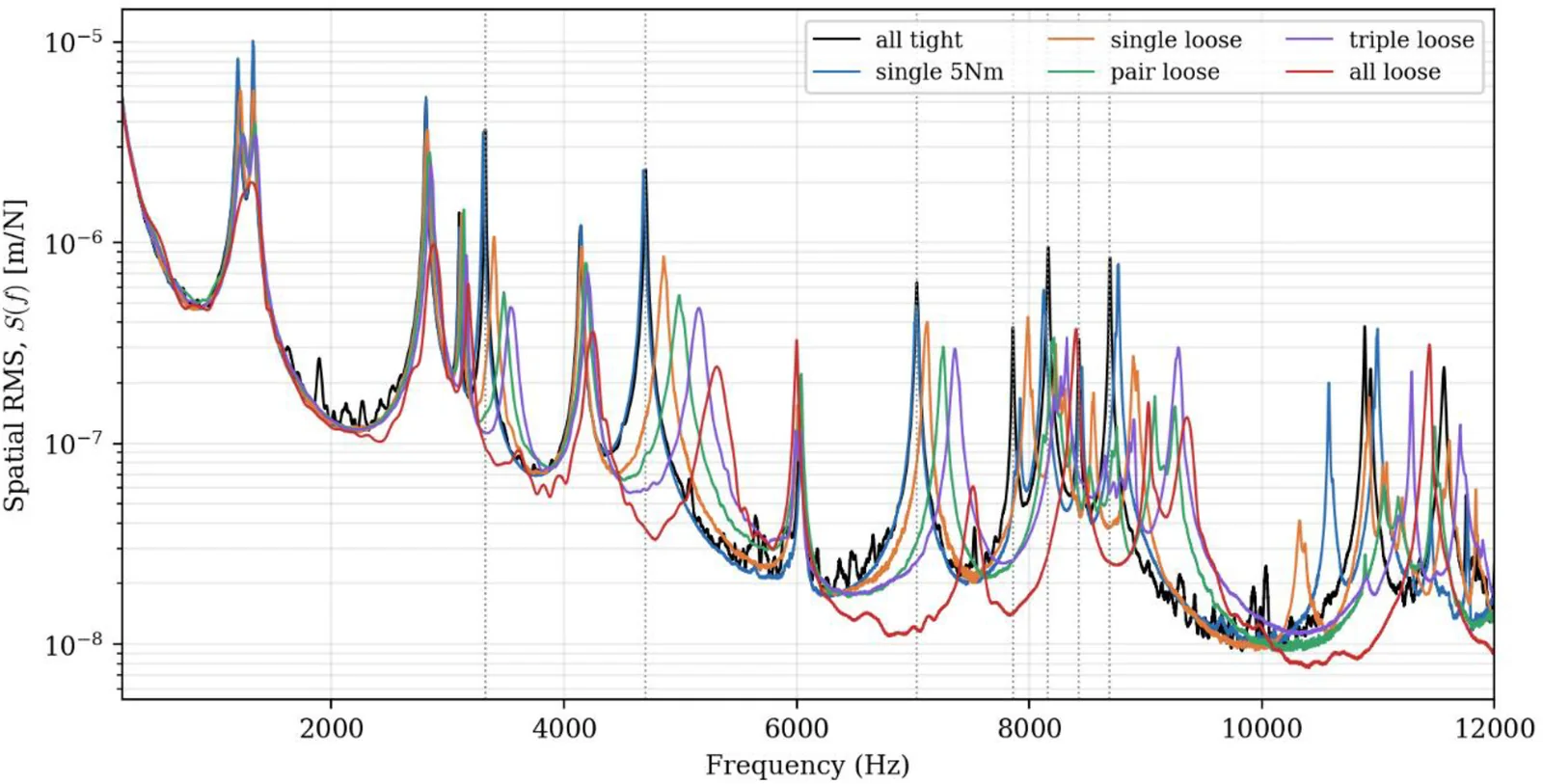
Spatial-RMS spectra across the 18 torque states (median per torque-state class) from 1 Hz to 12 kHz. The retained resonance groups are marked by vertical dotted lines.

**Fig. 4:**
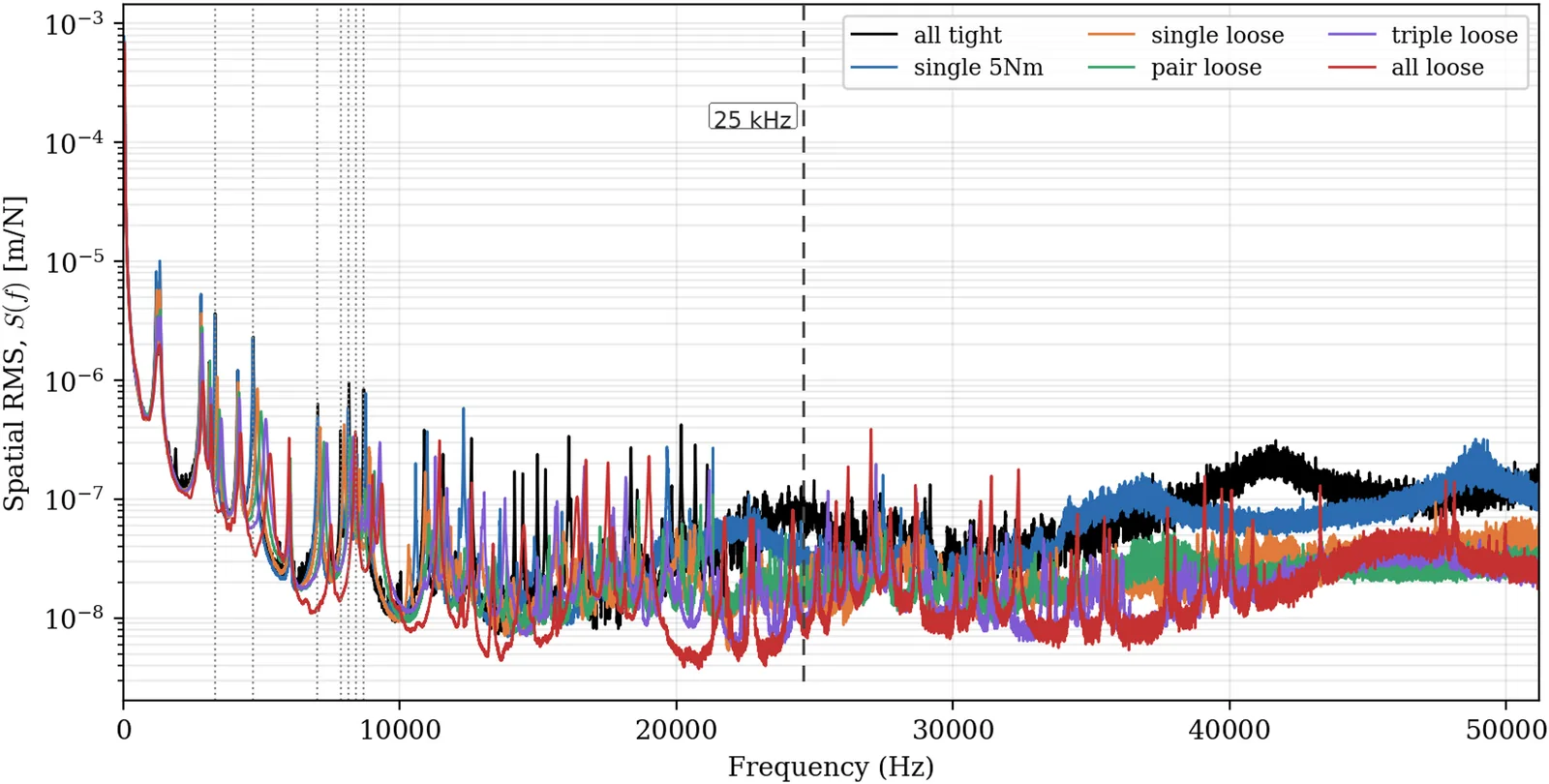
Spatial-RMS spectra across the 18 torque states (median per torque-state class) from 1 Hz to 51.2 kHz. The vertical dashed line at 25 kHz marks the recommended upper limit for quantitative FRF/correlation analysis for this specimen; the higher-frequency portion is retained in the raw exports but is dominated by a comparatively low signal level.

**Fig. 5:**
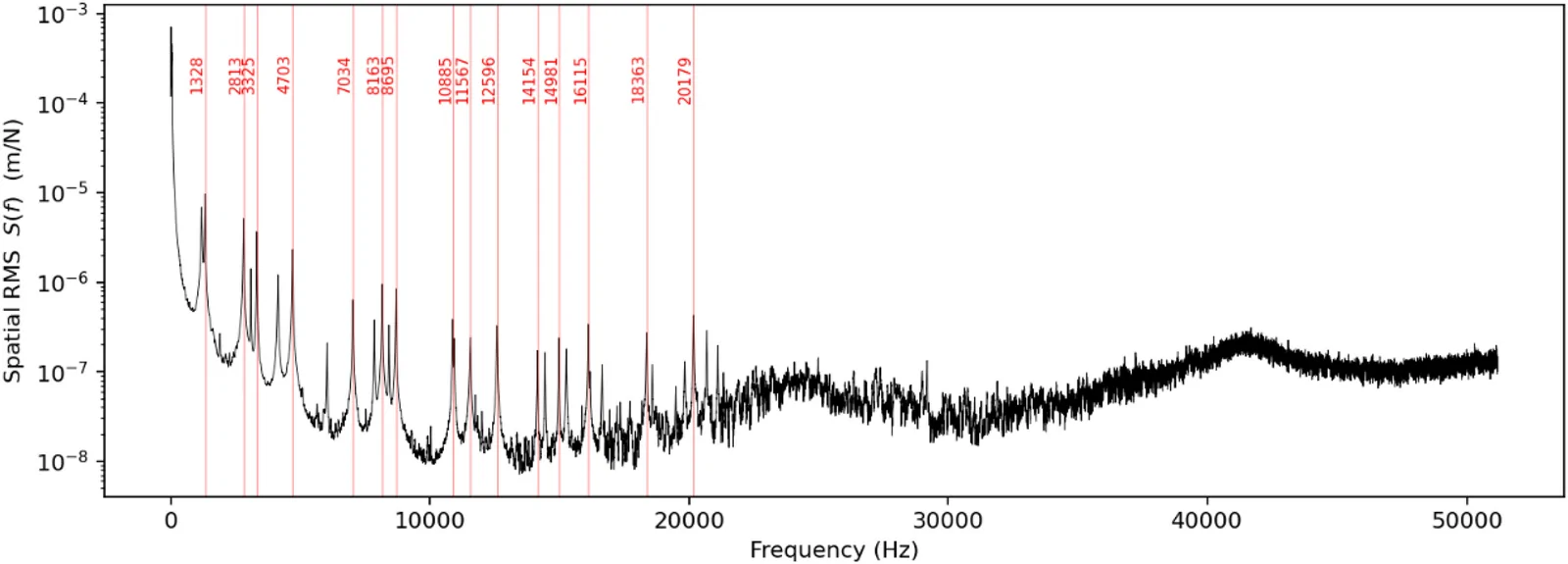
All-tight reference spatial-RMS spectrum with the picked candidate resonance peaks marked by red vertical lines. The retained resonance groups are documented numerically in the retention-audit and adaptive-tracking-window tables.

**Fig. 6:**
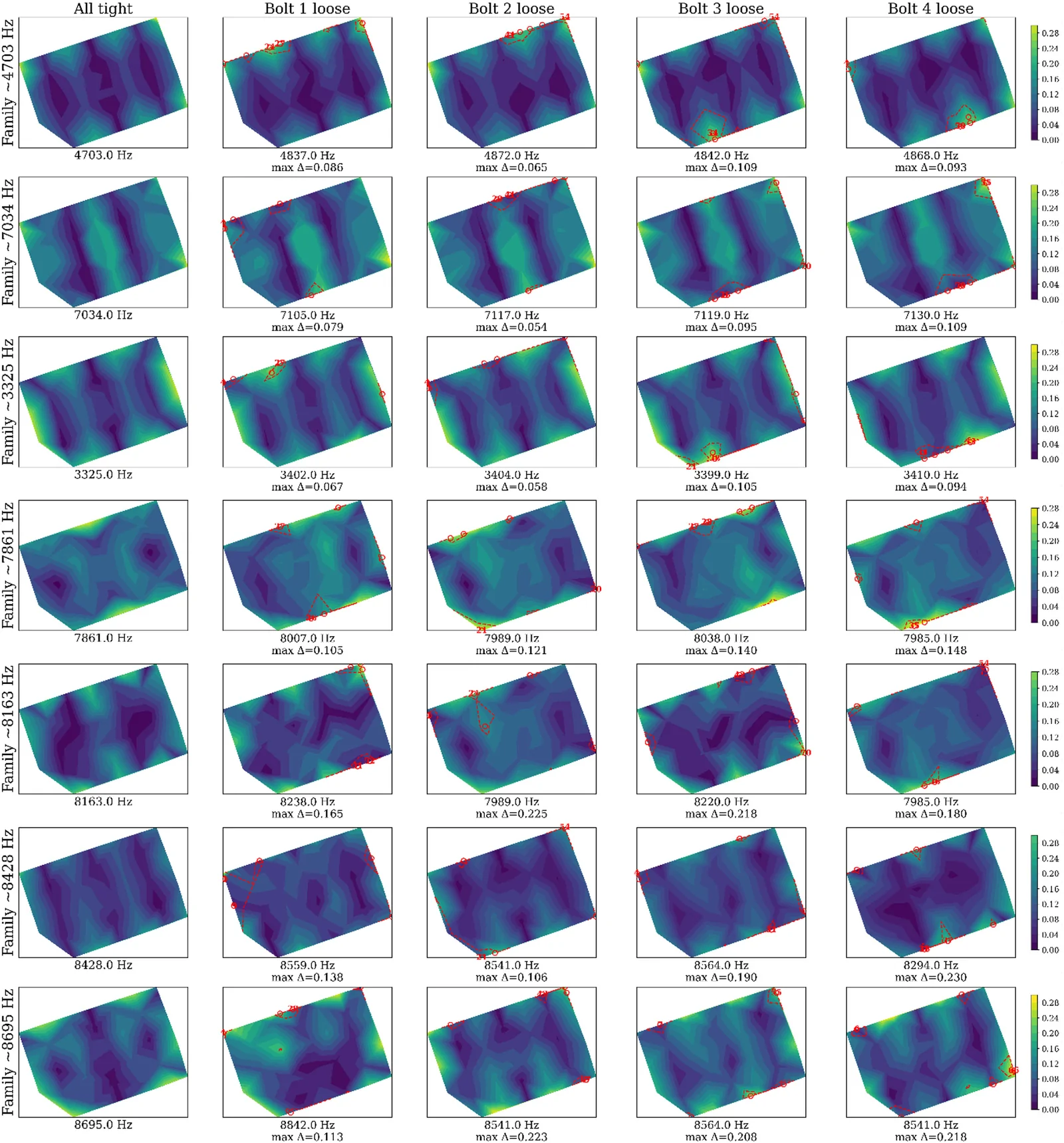
Local FRF-correlation sensitivity maps for the retained frequency groups (families), comparing the all-tight baseline with the four single-bolt-loose cases.

Table 1 lists the top-level folders. Table 2 summarizes the processed tabular datasets that a secondary user is most likely to inspect first. Table 3 is a compact variable dictionary for the main processed variables. Table 4 lists the eighteen torque configurations represented in the raw exports. Table 5 summarizes the dominant file types stored in the package.

**Table 1:** Top-level folder layout.

top_level_folder	purpose	n_files
00_raw_exports	Pointwise amplitude and phase H1 FRF TXT exports for the 18 torque configurations.	1838
01_documentation	Setup photograph, raw-export format note, scan-point coordinate list, torque-state metadata, and folder README.	5
02_processed_tables	Seven processed CSV tables plus folder README.	8
03_figures	Six PNG figures used in the data article plus folder README.	7
04_scripts	Six Python scripts plus folder README for rebuilding processed tables, figures, and optional HDF5 export.	7
05_quality_control_coherence	919 coherence spectra, recovered acquisition settings, QC summary, and checksums.	923
06_quality_control_force	919 Reference-1 force spectra, QC summary, and checksums.	922
metadata	DataCite and Schema.org machine-readable metadata files.	2

**Table 2:** Key processed tabular datasets.

file_name	content	rows	columns	key_variables
candidate_resonance_groups.csv	Discovery-set candidate peaks derived from the all-tight spatial-RMS spectrum.	15	4	$f_{\text{peak}}$; $\text{lo}g_{10}\left(p\right)$; $BW_{\text{HP}}$; $\Delta f_{\text{excl}}$
resonance_group_selection_audit.csv	Audit table documenting why candidates were retained or excluded.	15	8	rank(p) $f_{\text{peak}}$; r; $\Delta f_{\text{win}}$; $\text{rul}e_{\text{ret}}$
tracked_frequencies.csv	Tracked frequencies of the seven retained resonance groups across the all-tight and four single-bolt-loose cases.	7	6	$f_{\text{family}}$; $f_{\text{tight}}$; $f_{\text{bolt}1},\;\ldots ,\;f_{\text{bolt}4}$
per_case_metrics.csv	Case-wise amplitude-only, phase-aware, and phase-only global dissimilarity metrics.	28	7	g; b $f_{\text{base}}$; $f_{\text{case}}$; $1-\text{MA}C_{a}$; 1−CMAC; $1-\text{CMA}C_{\text{phase}}$
family_summary.csv	Family-wise mean, 95% exhaustive-bootstrap interval, extrema, and compactness summary metrics.	7	17	$f_{\text{family}}$; $\bar{1-\text{MA}C_{a}}$; $\bar{1-\text{CMAC}}$; $\bar{C_{5}}$; $\bar{R_{5}}$
dose_response.csv	Per-bolt 10/5/0 Nm dose-response of the global metrics for every retained resonance group.	84	7	g; b; T; $f_{\text{tracked}}$; $1-\text{MA}C_{a}$; 1−CMAC; $1-\text{CMA}C_{\text{phase}}$
adaptive_tracking_windows.csv	Tracking-window definitions for retained resonance groups.	7	6	$f_{\text{ret}}$; $BW_{\text{HP}}$; $\Delta f_{\text{cand}}$; $\Delta f_{\text{win}}$

**Table 3:** Variable dictionary for the main processed variables.

variable	units	description
$f_{\text{peak}}$	Hz	Center frequency of a discovery-set candidate peak.
$\text{lo}g_{10}\left(p\right)$	log10 scale	Peak prominence on the smoothed spatial-RMS spectrum.
$BW_{\text{HP}}$	Hz	Half-power bandwidth of a candidate or retained resonance group.
r	Boolean	True if the candidate passed the unique-trackability rule.
$\Delta f_{\text{win}}$	Hz	Rounded half-width of the tracking window assigned to a retained group.
$f_{\text{tight}}$	Hz	Tracked resonance frequency in the all-tight baseline case.
$f_{\text{bolt}1},\;\ldots ,\;f_{\text{bolt}4}$	Hz	Tracked resonance frequencies in the four single-bolt-loose cases.
$1-\text{MA}C_{a}$	dimensionless	Case-wise amplitude-only global dissimilarity.
1−CMAC	dimensionless	Case-wise phase-aware global dissimilarity.
$1-\text{CMA}C_{\text{phase}}$	dimensionless	Case-wise phase-only complex similarity (CMAC of unit-modulus phase pattern).
T	Nm	Bolt torque level (10, 5, or 0) for the dose-response analysis.
$\bar{C_{5}}$	dimensionless	Family-wise mean compactness score C5 for local FRAC-deficit maps.

**Table 4:** Eighteen torque configurations exported in 00_raw_exports.

case_name	human-readable label	bolt torques (Nm) for bolts 1, 2, 3, 4
1234_10Nm	all tight (reference)	10, 10, 10, 10
1234Lose	all loose	0, 0, 0, 0
234_10Nm_1Lose	bolt 1 loose	0, 10, 10, 10
234_10Nm_1_5Nm	bolt 1 at 5 Nm	5, 10, 10, 10
134_10Nm_2Lose	bolt 2 loose	10, 0, 10, 10
134_10Nm_2_5Nm	bolt 2 at 5 Nm	10, 5, 10, 10
124_10Nm_3Lose	bolt 3 loose	10, 10, 0, 10
124_10Nm_3_5Nm	bolt 3 at 5 Nm	10, 10, 5, 10
123_10Nm_4Lose	bolt 4 loose	10, 10, 10, 0
123_10Nm_4_5Nm	bolt 4 at 5 Nm	10, 10, 10, 5
1_10Nm_234Lose	bolts 2, 3, 4 loose	10, 0, 0, 0
2_10Nm_134Lose	bolts 1, 3, 4 loose	0, 10, 0, 0
3_10Nm_124Lose	bolts 1, 2, 4 loose	0, 0, 10, 0
4_10Nm_123Lose	bolts 1, 2, 3 loose	0, 0, 0, 10
13_10Nm_24Lose	bolts 2, 4 loose (diagonal pair)	10, 0, 10, 0
24_10Nm_13Lose	bolts 1, 3 loose (diagonal pair)	0, 10, 0, 10
14_10Nm_23Lose	bolts 2, 3 loose (adjacent pair)	10, 0, 0, 10
23_10Nm_14Lose	bolts 1, 4 loose (adjacent pair)	0, 10, 10, 0

**Table 5:** Dominant file types in the package.

extension	description	n_files
txt	Original pointwise H1 amplitude/phase exports plus acquisition-quality coherence and Reference-1 force spectra.	3676
csv	Documentation codebooks and processed analysis tables.	9
png	Setup photograph and data-article figures.	7
py	Analysis, figure-rebuild, and HDF5-export scripts.	6

## 4 Experimental Design, Materials and Methods

The specimen is a 100 × 150 × 7 mm aluminum plate with four diagonally positioned M6 bolts and an additional unused M6 hole at its center. The plate was supported on soft sponges to approximate, rather than reproduce, ideal free-free boundary conditions. A automatic modal hammer fitted with a steel tip excited one fixed point near the lower-left plate edge, close to bolt 3; a dynamic force sensor at the hammer tip provided the Reference-1 force signal. A Polytec PSV-500 scanning laser Doppler vibrometer measured the structural response non-contact. Each of the 18 nominal torque configurations in Table 4 is represented by one complete scan realization; independent torque-reset/reassembly repetitions of the complete scan were not archived. The 5 and 10 N m conditions were applied with a HAZET 5108B-3CT torque wrench (2.5–25 N m range; manufacturer-specified accuracy ±3% of scale value for right-hand tightening). The 0 N m states denote fully loosened bolts rather than a torque-wrench setting. The exact tightening sequence, experiment-specific wrench serial number/calibration certificate, and independent bolt-preload measurements are not archived; the tabulated torque values should therefore be interpreted as nominal control settings rather than direct preload measurements. The soft supports provided partial mechanical decoupling from the support table, and LDV avoided sensor mass loading. No dedicated active vibration-isolation or background-vibration subtraction protocol was documented, so environmental variability was not quantified separately.

The same PSV acquisition configuration was used across all 18 scans. The scan metadata record PSV software version 10.3.1.0. Data acquisition was performed in FFT mode with complex averaging over three averages, 51,200 FFT lines, a 128 kHz sampling frequency, a 1 s acquisition time, and 1 Hz frequency resolution over 1–51.2 kHz. Triggering used Reference 1 on a rising edge at a 1% threshold with a 0.5% pretrigger; automatic remeasurement and AutoRange were enabled. The vibrometer channel used a rectangular window and no acquisition-side filter. The force-reference channel used the PSV force window with parameter 0.507 and no acquisition-side filter; Reference 1 was operated at a 1 V range with AC coupling, 1 MΩ input impedance and IEPE enabled, with a calibration factor of 41.89359028 N/V. The vibrometer channel was acquired as velocity, and PSV integrated the response to displacement before forming/exporting the H1 displacement-to-force receptance FRF. Because the scans were stored in FFT mode, the time-domain force histories and the exact peak/target impact force cannot be recovered from the archived scans. The raw TXT amplitude files report the magnitude of the H1 displacement-to-force receptance in m/N, while phase files report the corresponding phase angle in degrees. Each file contains a seven-line Polytec header followed by tab-separated frequency and value columns. The amplitude header identifies the signal as “FFT - Vib & Ref1 H1 Weg / Kraft – Amplitude” (displacement divided by force). For each LDV scan point, the exported amplitude can be interpreted as given in Eq. (1):

(1) $$\left|H_{1}\left(f\right)\right|=\left|\frac{x\left(f\right)}{F\left(f\right)}\right|,$$

with units given in Eq. (2):

(2) $$\left[H_{1}\left(f\right)\right]=m/N$$

Here, x_i(f) is the displacement response at frequency f and F(f) is the reference force input. The phase TXT exports correspond to the phase of the same H1 displacement-to-force FRF. The raw phase values are stored in degrees, not radians. Any complex-valued FRF reconstruction must convert the phase to radians before applying the complex exponential. The phase header identifies the signal as “FFT - Vib & Ref1 H1 Weg / Kraft – Phase” (displacement divided by force). The complex FRF for scan point i is therefore reconstructed as given in Eq. (3):

(3) $$H_{i}\left(f\right)=A_{i}\left(f\right)\text{exp}\left(j\frac{\pi }{180}\phi_{i}\left(f\right)\right),$$

where A_i(f) is the exported amplitude in m/N and φ_i(f) is the exported phase in degrees at LDV point i.

The original H1 inventory contains 1838 text files: 919 amplitude exports and 919 phase exports. The all-loose state contains one additional exported point, so the main analysis script intersects the available point indices across all 18 torque states and both signal types, preserving the 51 coordinate points present everywhere. The script then calculates the all-tight spatial root-mean-square spectrum, detects candidate peaks from the smoothed log spectrum, evaluates bandwidth/spacing information and the documented retention criterion, defines tracking windows for retained groups, tracks frequencies, and computes the global and local correlation-based products. Its outputs are organized into candidate-resonance tables, retention-audit tables, adaptive tracking-window tables, tracked-frequency matrices, case-wise global correlation measures, discrete 10/5/0 N m torque-level tables, and family-wise summary measures. These global and local measures map onto established MAC, FRAC, and FRF-shape localization concepts [[10], [11], [12], [13]]. The repository file name dose_response.csv is retained for compatibility, but the table contains only three discrete nominal torque levels and should not be interpreted as a continuous dose-response function. The supplementary quality-control layer is split between 05_quality_control_coherence (complete 919-spectrum coherence layer) and 06_quality_control_force (complete 919-spectrum Reference-1 force layer); these diagnostic signals are documented separately and are not used to redefine the H1 processing pipeline.

Secondary analyses can be conducted in two modes. Users may directly reuse the processed CSV tables and figures, or they may start from 00_raw_exports and run the scripts in 04_scripts to reproduce or modify the processed layer. Parsing and variable definitions are documented in the repository documentation, including the raw-export format note and data dictionary. Acquisition-quality checks from the original PSV scans show very high coherence at the seven retained all-tight resonance groups: across the 51 all-tight scan points, median coherence at 3.325–8.695 kHz ranges from 0.999748 to 0.999977. Across the 918 common point-state Reference-1 force spectra, the pointwise median force spectrum is approximately 18.6 dB below its median 1–5 kHz level at 20 kHz and approximately 26.5 dB below that reference at 25 kHz; across the corresponding 918 common point-state coherence spectra, the median coherence at 25 kHz is approximately 0.249. These diagnostics support the recommended usable range of 1 Hz to approximately 25 kHz for quantitative FRF/correlation analysis of this specimen and show progressive deterioration toward the upper boundary. The 25–51.2 kHz portion is retained for completeness rather than treated as equally informative resonance content. The package is intended to follow the FAIR data principles [14] and to be cited as a research output in line with the Joint Declaration of Data Citation Principles [15].

## Limitations

This dataset represents one aluminum plate, one material and geometry, one four-bolt layout, one fixed excitation point, one soft-support realization, and one complete scan realization per nominal torque state. Generalization to other plate dimensions, materials, joint geometries, or boundary conditions therefore requires independent validation. The sponge-supported condition is only an approximation of ideal free-free behavior; the supports can introduce additional stiffness and damping. Likewise, single-point hammer excitation can weakly excite modes having low mobility at the impact position, which can influence FRF-based and correlation-oriented analyses. The available nominal torque levels are discrete (10, 5, and 0 N m) and cannot characterize a continuous torque/preload transition. Direct preload measurements, the exact tightening sequence, and the experiment-specific torque-wrench serial number/calibration certificate are not available. Although the FFT acquisition settings, averaging, windowing, filtering and force-channel calibration were recovered from the PSV scan metadata, the scans do not preserve the time-domain impact-force histories or exact peak/target impact force. No repeated specimens, complete torque-reset scan repetitions, matched finite-element simulations, cyclic-loading tests, or long-term ageing tests are included; the dataset is therefore not intended for fatigue-, creep-, or ageing-related inference. The raw export band extends from 1 Hz to 51.2 kHz, but the quality-control coherence and force spectra confirm deteriorating signal quality toward the upper band; quantitative FRF/correlation analysis is therefore recommended only up to approximately 25 kHz for this specimen. Finally, raw phase values are reported in degrees and must be converted to radians before complex-FRF reconstruction.

## Ethics Statement

The authors have read and follow the ethical requirements for publication in Data in Brief. The work does not involve human subjects, animal experiments, or data collected from social media platforms.

## CRediT Author Statement

**Berkay Kullukcu:** Conceptualization, Methodology, Software, Data curation, Formal analysis, Visualization, Writing – Original draft. **Dina Hannebauer:** Supervision, Resources, Validation, Writing – Review & editing.
